# O2.2. CHILDHOOD ADVERSITIES AND PSYCHOTIC SYMPTOMS: THE POTENTIAL MEDIATING OR MODERATING ROLE OF NEUROCOGNITION AND SOCIAL COGNITION

**DOI:** 10.1093/schbul/sby015.192

**Published:** 2018-04-01

**Authors:** Giovanni Mansueto, Koen Schruers, Fiammetta Cosci, Jim van Os, GROUP Investigators

**Affiliations:** 1 School for Mental Health and Neuroscience Maastricht University; 2 Maastricht University Medical Center, School for Mental Health & Neuroscience; 3 Florence University; 4 University of Maastricht

## Abstract

**Background:**

Childhood abuse and neglect are risk factors for psychotic symptoms. The exposure to early adversities might lead to poor functioning in the area of neuro/social cognition, which in turn is associated to psychosis. This study aimed to explore the mediating and moderating role of neuro/social cognition in the relationship between childhood abuse, neglect, and psychotic symptoms.

**Methods:**

1.119 psychotic patients were enrolled from university hospitals in the Netherlands and Belgium (i.e., Groningen, Amsterdam, Maastricht, Utrecht, Leuven) and their affiliated mental healthcare institutions. Childhood adversities were evaluated with the Dutch version of the Childhood Trauma Questionnaire. Psychotic symptoms were assessed with the Positive and Negative Syndrome Scale, using the five-factor model proposed by van der Gaag et al. (2006). Verbal learning-memory, attention-vigilance, working memory, information processing speed, reasoning-problem solving were evaluated via the Word Learning Task, the Continuous Performance Test, the Wechsler Adult Intelligence Scale 3rd. Mentalizing abilities were evaluated as a measure of social cognition using the Hinting Task. Mann-Whitney U test were performed to compare patients with to without early adversities. Correlation was used to ensure that independent variables (childhood neglect or childhood abuse), dependent variables (psychotic symptoms), and hypothesized mediator (M)/moderator (MR) (i.e., neurocognition or social cognition) were associated. Mediation and moderation analyses were run according to Baron and Kenny’s criteria. A bootstrapping procedure was used to assess indirect effects. Mediation and moderation models were adjusted for age, sex, and lifetime cannabis use as a priori potential cofounders

**Results:**

Patients with childhood neglect, compared with those without childhood neglect, showed more severe psychotic symptoms (p<.01) and lower scores on retention rate, and attention (p<.01).

Patients with childhood abuse showed more severe psychotic symptoms (p<.01) than those without childhood abuse, while no statistically significant differences were found for neurocognition (p=.87) and social cognition (p=.77).

Mentalizing abilities partially mediate the relationship between childhood neglect and negative symptoms (Total Effect: 1.01, BCa: .27-.75; Indirect effect: .17, BCa: .02-.40), disorganization (Total Effect: 1.29, BCa .46-1.95; Indirect effect: .23, BCa: .03-.49), and excitement (Total Effect: .76, BCa: .32-1.21; Indirect effect: .05, BCa: .009-.14). The association between childhood neglect and psychotic symptoms is neither mediated nor moderated by neurocognition. Neurocognition and social cognition neither mediate nor moderate the association between childhood abuse and psychotic symptoms.

**Discussion:**

The aetiological role of neurocognition in the association between childhood adversities and psychosis seems unlikely. Mentalizing abilities could be an aetio-pathogenetic pathway linking childhood neglect to negative symptoms, disorganization, and excitement.

